# A Quad-Channel Diplexer Using Stub-Loaded Step Impedance Resonators

**DOI:** 10.3390/mi16091012

**Published:** 2025-08-31

**Authors:** Liqin Liu, Zhenheng Lin, Qun Chen, Li Zhang, Minhang Weng, Ruyuan Yang

**Affiliations:** 1 College of Artificial Intelligence, Electronic Information Industry Technology Research Institute of Putian, Putian University, Putian 351100, China; 2 Graduate Institute of Materials Engineering, National Pingtung University of Science and Technology, Pingtung City 912, Taiwan

**Keywords:** quad-channel, diplexer, dual-band, high isolation

## Abstract

A quad-channel diplexer is designed in this paper. The diplexer is composed of four stub-loaded step impedance resonators and a common feeder T-joint, which realizes four passbands with center frequencies of 2.6 GHz, 3.48 GHz, 4.8 GHz, and 6.3 GHz. The dual-band filter can be formed by coupling the stepped impedance resonator with the stub load, so two dual-band filters with good performance can be constructed. At the input and output end, a 0-degree feed is used to generate transmission zeros, which improves the high selectivity. When two dual-band filters are combined, a good impedance matching is obtained, and the |S_23_| > 20 dB between the two dual-band filters achieves good isolation. The simulation results are consistent with the measured results.

## 1. Introduction

With the continuous development of communication technology and the need to process the growing mass of data, the development of new band-pass filtering structure has been greatly promoted. In parallel with multiband bandpass filters, diplexers are also widely utilized in RF front-end systems to divide/combine two or more frequency channels [1,2,3,4,5]. Therefore, the study of high-performance diplexer is also an important research topic.

Diplexer/multiplexer components have been widely used in RF/microwave front-end systems to realize the separation of two or more signals of different frequency bands in the same channel without mutual interference [6]. There have been many efforts to study diplexers and multiplexers [7,8,9,10,11,12,13,14,15]. Songyao Ji et al. [7] designed a quad-channel diplexer using a C-section impedance matching circuit. Each C-section impedance matching circuit can independently control one passband in the diplexer. The center frequencies of the quad-channel diplexer are 0.9/1.23/2.4/3.5 GHz. It was successfully realized. Baoping Ren et al. [8] designed a stub-loaded ring resonator with shorted points. Different resonators A and B formed by the shorted points at different locations on the resonator and the resonance properties of resonators A and B were investigated, and finally a quad-channel diplexer was designed using resonators A and B. Ali K. Gorur et al. [9] designed a quad-channel diplexer by coupling two sets of isotropic split-ring resonators, which had good isolation performance but the insertion loss of the various passbands was a bit large. Sobhan Roshani et al. [10] designed a four-channel diplexer by using triangular loop resonators and rectangular loop resonators. This diplexer has a relatively small volume and low insertion loss, but its structure is rather complex. Over the course of the research, some scholars have utilized stub-loaded step impedance resonators to design a quad-channel diplexer [11,12]. Sugchai TANTIVIWAT et al. [12] realized the conversion of a three-mode resonator into a four-mode resonator by introducing an intercoupled line resonance between the odd modes in a three-mode stub-loaded step impedance resonator and designed a quad-channel diplexer. The diplexer achieved good isolation performance, but the insertion loss of each passband was also a bit large. Some scholars have also investigated balanced quad-channel diplexers [13,14,15]. Wen Tao Li et al. [15] designed a new unbalanced-to-balanced quad-channel diplexer consisting of two balun filters sharing a single-ended microstrip feeder. The structure was complex and also the insertion loss of each passband was high. As a result, designing quad-channel diplexers with good performance such as small circuit size, low insertion loss, high isolation, and high selectivity remains a challenge.

In this paper, a highly isolated diplexer is proposed to achieve four passbands which center frequencies are 2.6 GHz, 3.48 GHz, 4.8 GHz, and 6.3 GHz.2.6 GHz is used for 4G communication system, 3.48 GHz and 4.8 GHz are used for a 5G communication system, 6.3 GHz is used in satellite communication systems. The detailed implementation steps are as follows:

(1) Stub-loaded step impedance resonators (SLSIRs) are analyzed to realize miniaturized BPFs to meet the passband requirements. In addition, by analyzing the odd–even mode, the position of the transmission zero is accurately predicted, and the high-performance BPF is realized, which has a sharp transition band.

(2) Two BPFs are designed at 3.48/6.3 GHz and 2.6/4.8 GHz to achieve the ideal performance diplexer.

(3) The two BPFs are merged. High isolation can also be achieved between the four channels because of the good impedance matching at the input.

The study details the proposal, design, manufacture, and testing of diplexer. The measurement results show that the prepared quad-channel diplexer is in good agreement with the simulation results.

## 2. Design Process

**Figure 1:**
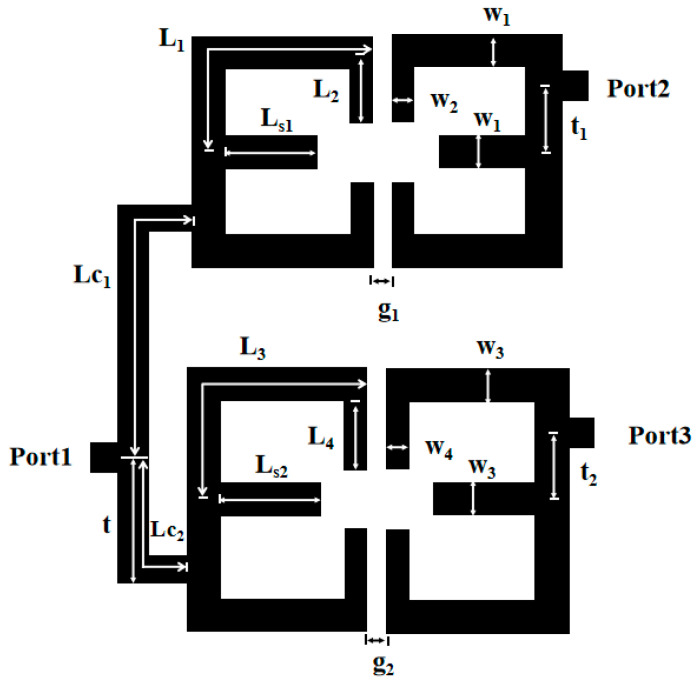
Design structure of a quad-channel diplexer filter.

Figure 1 is the design structure of a quad-channel diplexer in this paper. The filter in the diplexer is implemented by two electrically coupled SLSIRs. The upper dual-band filter implements 2.6/4.8 GHz; the lower dual-band filter implements 3.48/6.3 GHz. In the paper, the diplexer, filter, and resonator are all using 5880 substrates with a dielectric parameter of 2.2, a substrate thickness of 0.787 mm, and a loss tangent constant of 0.0009.

### 2.1. Stub Loaded Step Impedance Resonator(SLSIR) Characterization

**Figure 2:**
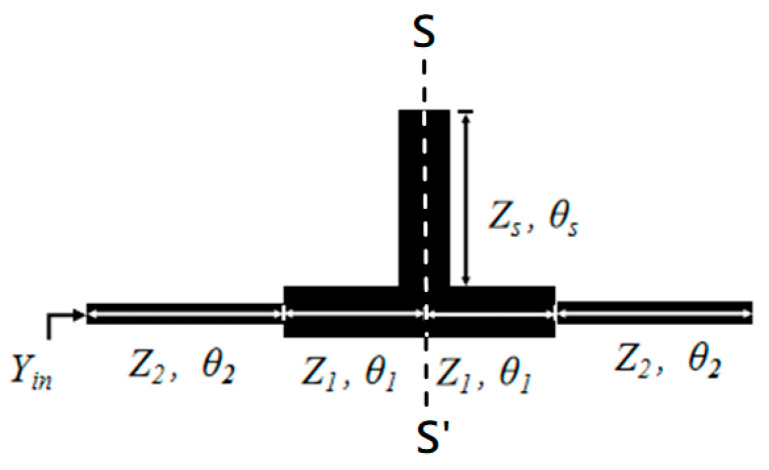
The structure of the Stub Loaded Step Impedance Resonator(SLSIR).

The Stub Loaded Step Impedance Resonator(SLSIR) resonator adopted in the article is shown in Figure 2. It is loaded on an SIR by a stub which is located on the line of symmetry (S-S′) of the SIR. In the SLSIR structure, Z_1_ represents the low impedance, *θ*_1_ represents the electrical length corresponding to the low impedance; Z_2_ represents the high impedance, *θ*_2_ represents the electrical length corresponding to the high impedance, Z_s_ represents the impedance of the stub, and *θ*_s_ represents the electrical length corresponding to the stub. Analyzing the resonant characteristics of the SLSIR is a necessary condition for understanding the frequencies of various resonant modes. For the convenience of analysis, two impedance ratios are defined as K_1_ = Z_2_/Z_1_ and K_2_ = Z_s_/Z_1_. Further, the input admittance Yin of the SLSIR is derived. The solution satisfying the condition of Y_in_ = 0 is the resonant mode of the SLSIR. 

**Figure 3:**
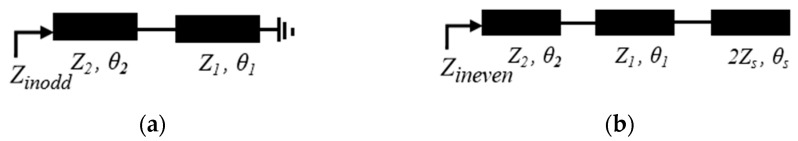
(**a**) The structure of the odd-mode resonance in the SLSIR; (**b**) the structure of the even-mode resonance in the SLSIR.

However, since the SLSIR used is symmetrical, the odd–even mode analysis method can be adopted to analyze the resonant characteristics. In the odd-mode resonance, the symmetry plane of the SLSIR is an electric wall; in the even-mode resonance, the symmetry plane of the SLSIR is a magnetic wall. Figure 3a shows the structure of the odd-mode resonance in the SLSIR and (b) shows the structure of the even-mode resonance in the SLSIR. According to the transmission theory, the resonant condition corresponding to the odd-mode and even-mode resonance of the SLSIR can be obtained as Equations (1) and (2):

(1) $$\left(K_{1}-\tan\theta_{1}\tan\theta_{2}\right)=0$$

(2) $$\left[2K_{2}\left(K_{1}\tan\theta_{1}+\tan\theta_{2}\right)+\tan\theta_{s}\left(K_{1}-\tan\theta_{1}\tan\theta_{2}\right)\right]=0$$

To better analyze the higher-order resonant modes of the resonator, two electrical length ratios of the discontinuity points of the SIR are defined as *α* = 2*θ*_2_/2(*θ*_1_ + *θ*_2_) = 2*θ*_2_/*θ*_T_, and the electrical length ratio of the stub section as *r* = 2*θ*_S_/*θ*_T_, where *θ*_T_ is the total length of the SIR section. To simplify the design, assume Z_s_ = Z_1_, that is, K_2_ = 1, and the resonant curves under different parameters K_1_, α, and r can be plotted. Substituting the formulas of the length ratios (α) and (*r*) into (1) and (2), the resonant conditions corresponding to the odd–even mode resonances as functions of α, *r*, and K_1_ are (3) and (4), respectively.

(3) $$K_{1}-\tan\left(\frac{1-\alpha }{2}\theta_{T}\right)\tan\left(\frac{\alpha }{2}\theta_{T}\right)=0$$

(4) $$2\left(K_{1}\tan\left(\frac{1-\alpha }{2}\theta_{T}\right)+\tan\left(\frac{\alpha }{2}\theta_{T}\right)\right)+\tan\left(\frac{r}{2}\theta_{T}\right)\left(K_{1}-\tan\left(\frac{1-\alpha }{2}\theta_{T}\right)\tan\left(\frac{\alpha }{2}\theta_{T}\right)\right)=0$$

**Figure 4:**
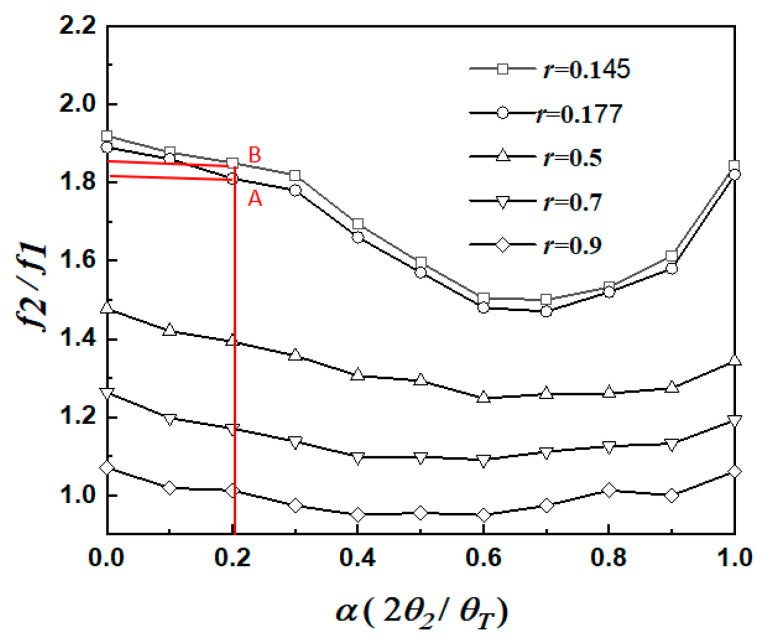
Resonance diagram of *f*_2_/*f*_1_ based on K_1_ = 2 and K_2_ = 1.

The solutions satisfying Equations (3) and (4) are related to the variables *α*, *r* and K_1_. Because the structure is very simple, which mainly composes a typical SIR with a stub loaded at the symmetric line, only the first passband can be independently controlled, and the higher-order modes basically change together with the first mode. The odd mode of the SLSIR resonator generates the fundamental frequency *f*_1_ of the resonator, and the even mode of the SLSIR resonator generates the frequency *f*_2_ of the second higher-order mode. Different K_1_ and K_2_ can be used to design dual-band filters. Figure 4 shows the *f*_2_/*f*_1_ resonance diagram drawn using the MATLAB tool when the SLSIR is based on K_1_ = 2 and K_2_ = 1, and at different length ratios *r* = 0.145, 0.177, 0.5, 0.7, and 0.9. When the electrical length ratio is *α* = 0.2 and *r* = 0.177, A is *f*_2_/*f*_1_ = 1.81; when the electrical length ratio is *α* = 0.2 and *r* = 0.145, B is *f*_2_/*f*_1_ = 1.85.

### 2.2. Two Dual-Band Filter Design Process

In the second step, in order to design the quad-channel diplexer, two dual-band filters need to be designed for the upper and the lower part, respectively.

(1) The center frequency of the upper part of the dual-band filter is 3.48 GHz and 6.3 GHz, and the −3 dB bandwidth is 17.8% and 13.5%, respectively, and a passband ripple of 1 dB. since *f*_2_/*f*_1_ = 1.75, according to Figure 4, the simulated filter parameters K_1_ = 2 and K_2_ = 1 are selected with *α* = 0.2 and *r* = 0.177. According to the designed filter performance parameters, the total set values of the corresponding Chebyshev low-pass prototype filter circuit elements are G_1_ = 1.8219 and G_2_ = 0.6850. And the theoretical coupling coefficient equation is as follows [16]:

(5) $$M_{i,j}=\frac{FBW}{\sqrt{G_{1}G_{2}}}$$

where FBW is the bandwidth, substituting G_1_ and G_2_ into the above equation, the theoretical coupling coefficients are obtained as 0.199 and 0.15; and the calculated coupling coefficients ($K_{i,j}$) are obtained through full-wave simulation responses under weak coupling conditions, and their expression is as follows [16]:

(6) $$K_{i,j}=\frac{{f_{H}}^{2}-{f_{L}}^{2}}{{f_{H}}^{2}+{f_{L}}^{2}}$$

where *f*_H_ is the high frequency and *f*_L_ is the low frequency in the two resonant modes, respectively.

**Figure 5:**
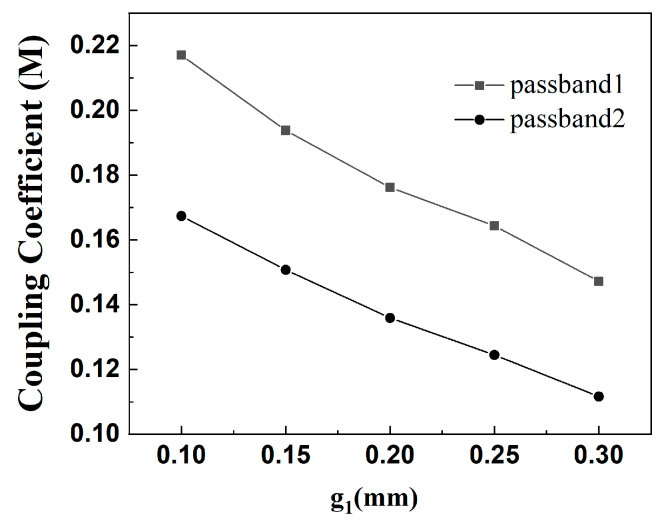
The upper part of the dual-band filter coupling coefficient diagram.

**Figure 6:**
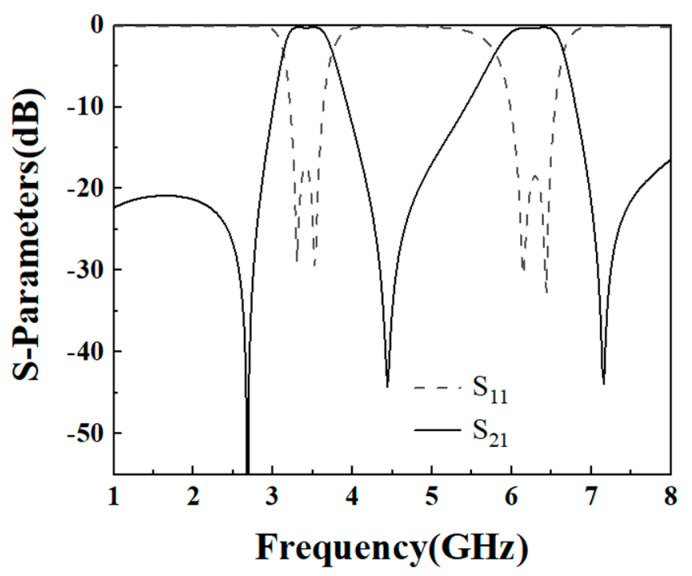
The upper part of the simulation effect of the dual-band filter.

In this paper, once the simulated filter parameters K_1_ = 2 and K_2_ = 1 are selected with *α* = 0.2 and *r* = 0.177 are determined, the prototype of the dual-band filter is established. To achieve the desired filter performance, the adjustable parameter is the gap g1. Figure 5 plots the calculated coupling coefficient of the upper part of the dual-frequency filter, from which it can be seen that when the coupling spacing *g1* is larger, the coupling coefficient of the two passbands is smaller. As the carving machine used in this paper can only realize the minimum spacing of 0.2 mm, so the coupling spacing is *g1* = 0.2 mm. Figure 6 shows the simulation effect of the upper part of the dual-frequency filter, which achieves the center frequencies of 3.48 GHz and 6.3 GHz, and the −3 dB bandwidths of 17.8% and 13.5%. Due to the use of the 0-degree feed-in method, three transmission zeros are obtained, and the dual-band filter is realized with high isolation and selectivity. Simulation parameters are as follows: L_1_ = 14.76 mm, L_2_ = 3.9 mm, L_s1_ = 3.3 mm, W_1_ = 1.6 mm, W_2_ = 0.36 mm, *g*_1_ = 0.2 mm; t_1_ = 4 mm, high impedance 130 ohms 0.36 mm, low impedance 65 ohms 1.6 mm, *α* = 0.2, *r* = 0.177.

**Figure 7:**
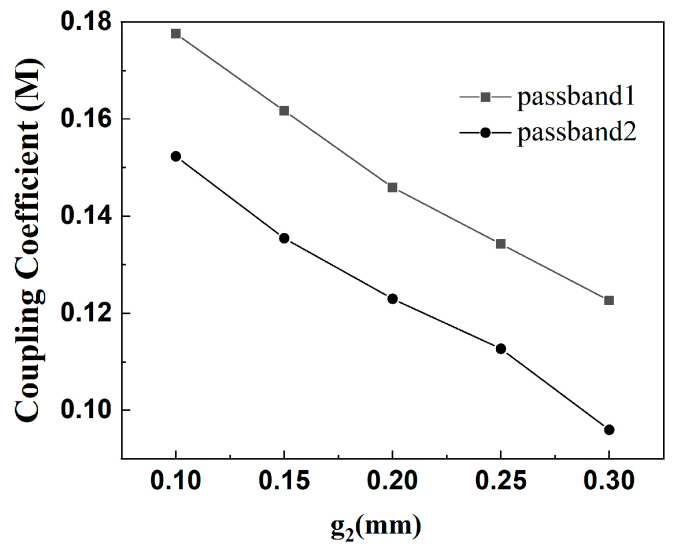
Plot of coupling coefficients of the lower part of the dual-band filter.

**Figure 8:**
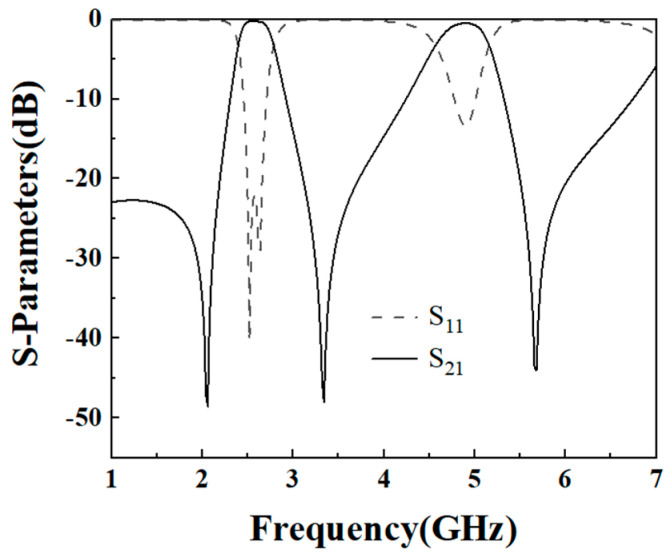
Simulation effect of the lower part of the dual-band filter.

(2) The lower dual-band filter has center frequencies of 2.6 GHz and 4.8 GHz, −3 dB bandwidths are 14.6% and 13.5%, respectively, and a passband ripple of 0.5 dB. Because *f*_2_/*f*_1_ = 1.85, according to Figure 7, the simulated filter parameters K_1_ = 2 and K_2_ = 1 are selected with α = 0.2 and r = 0.145. The lower dual-band filter design is similar to the upper dual-band filter design process. Figure 7 shows the coupling coefficients of the lower part of the dual-band filter, from which it can be seen that the coupling coefficients of the two passbands are smaller when the coupling spacing g2 is larger. Figure 8 shows the simulation effect of the lower part of dual-frequency filter. The center frequency is 2.6 GHz and 4.8 GHz, and the bandwidths of −3 dB are 14.6% and 13.5%. At the same time, three transmission zeros are obtained due to the adoption of the 0-degree feed method, which realizes the high isolation and selectivity of the dual-band filter. The simulation parameters are as follows: L_3_ = 19.45 mm, L_4_ = 4.7 mm, L_s2_ = 3.5 mm, W_3_ = 1.8 mm, W_4_ = 0.45 mm, g_2_ = 0.2 mm, t_2_ = 5 mm, K = 2.

### 2.3. Impedance Matching Diplexer Design

**Figure 9:**
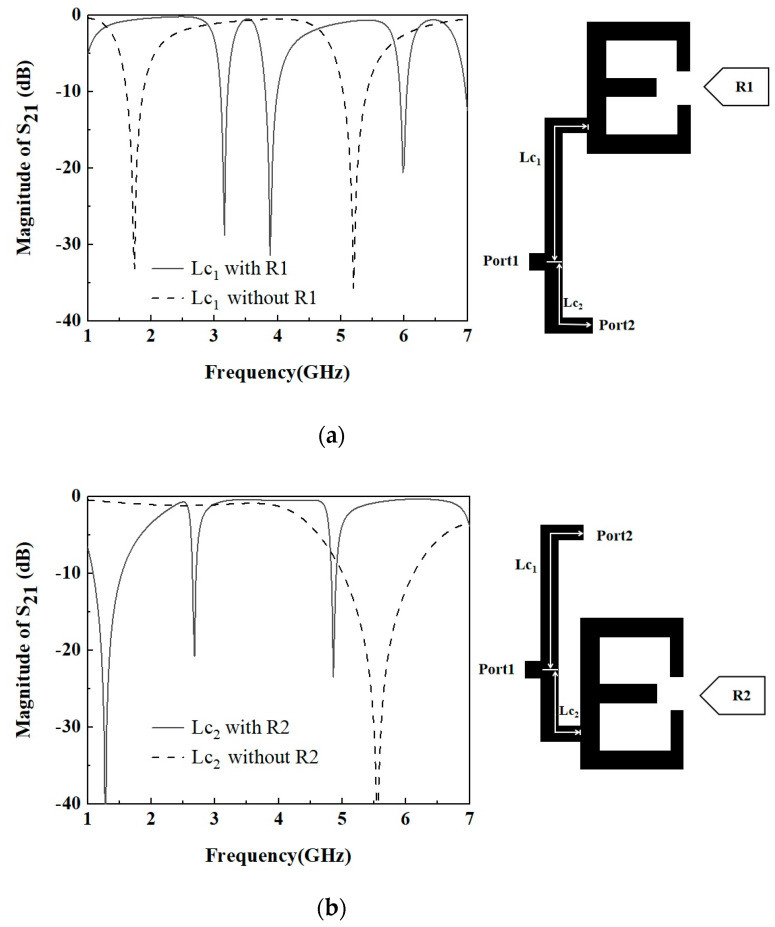
Simulated frequency response of the tap-branch structure coupled line as matched load. (**a**) Dual-band filter at 3.48 GHz and 6.3 GHz; (**b**) dual-band filter at 2.6 GHz and 4.8 GHz.

The third step is to combine two dual-band filters. The performance of a single dual-band filter can meet the requirements of 4G and 5G communication systems, 5G communication systems, and satellite communication systems, but when the two dual-band filters are combined to form a diplexer, the performance of a single bandpass filter may be distorted. In this case, impedance matching is needed to achieve the formation of a diplexer without distorting the performance of a single bandpass filter. In Figure 1, port 1 is the input port, while port 2 and port 3 are the output ports of the two BPFs. In order to realize the impedance matching of the diplexer, it needs to design the optimal coupling line length for the tap branch structure. In this paper, the length of the coupling lines (Lc_1_ and Lc_2_) of the tap branch structure is adjusted to achieve impedance matching. The necessary conditions for impedance matching of the diplexer are as follows: when the upper BPF works, the lower BPF sees an infinite impedance; when the lower BPF works, the upper BPF sees an infinite impedance. Through IE3D simulation, it is obtained that a good impedance matching is achieved when the length of the branch Lc_1_ = 32.1 mm and Lc_2_ = 10.1 mm. Figure 9 shows the simulated frequency response of a tap branching structure with or without load. In Figure 9a, the S21 shows the transmission from the common port to the lower filter with 2.6 GHz and 4.8 GHz passbands, the resonator R1 and transmission line with length of Lc1 should produce suppression at the lower frequency bands. The reverse is shown in Figure 9b.

**Figure 10:**
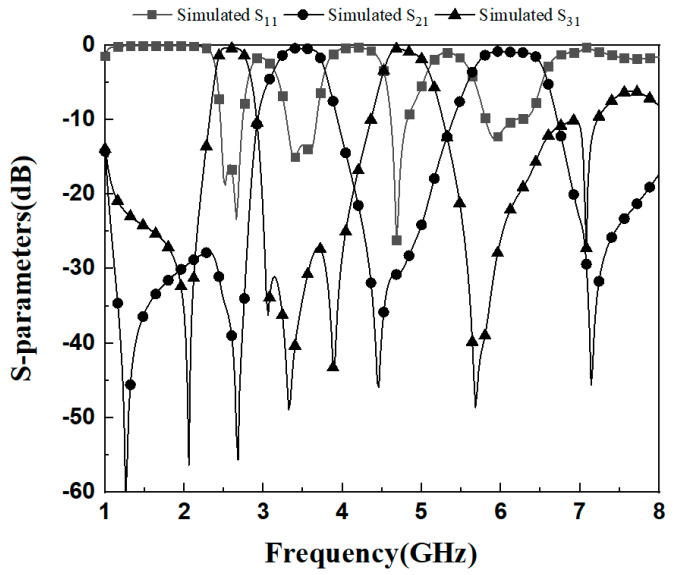
Simulation effect of diplexer simulation.

**Figure 11:**
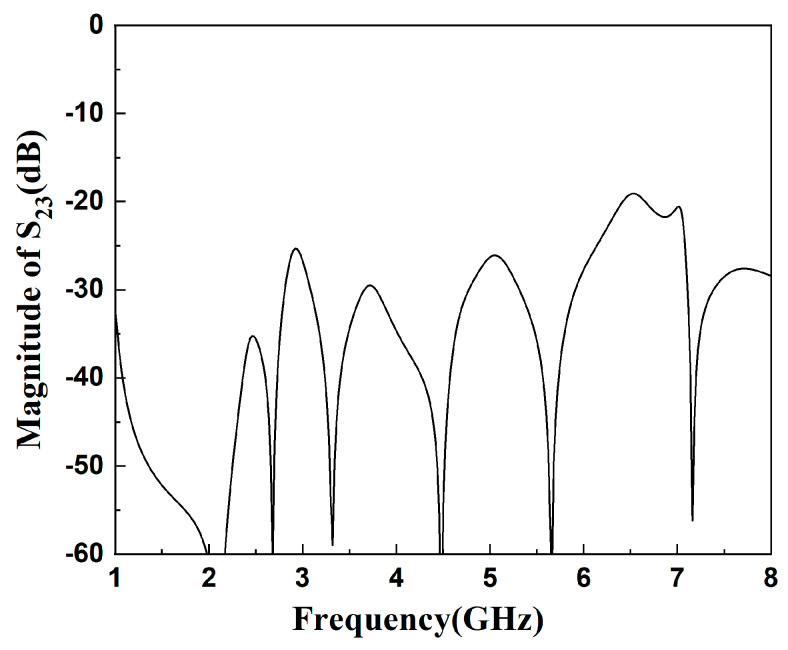
Parameter diagram of S_23_ in diplexer.

Figure 10 and Figure 11 show the simulation results and S_23_ parameters of the dual-band diplexer, respectively. The simulation parameters are as follows: L_1_ = 14.76 mm, L_2_ = 3.9 mm, L_s1_ = 3.3 mm, W_1_ = 1.6 mm, W_2_ = 0.36 mm, g_1_ = 0.2 mm; t_1_ = 4 mm, Z_2_ = 130Ω, Z_1_ = 65Ω, *α* = 0.2, *r*_1_ = 0.177; L_3_ = 19.45 mm, L_4_ = 4.7 mm, Ls_2_ = 3.5 mm, W_3_ = 1.8 mm, W_4_ = 0.45 mm, g_2_ = 0.2 mm; t_2_ = 5 mm, K = 2, Z_4_ = 120 Ω, Z_3_ = 60 Ω, r_2_ = 0.145; Lc_1_= 32.1 mm, Lc_2_ = 10.1 mm, t = 7.1 mm. This diplexer implements four channels as 3.48/6.3 GHz and 2.6/4.8 GHz. The simultaneous parameter |S_23_| > 20 dB indicates that the two dual-band filters in this diplexer have high isolation and the two dual-band filters do not interfere with each other.

## 3. Experimental Test Results

**Figure 12:**
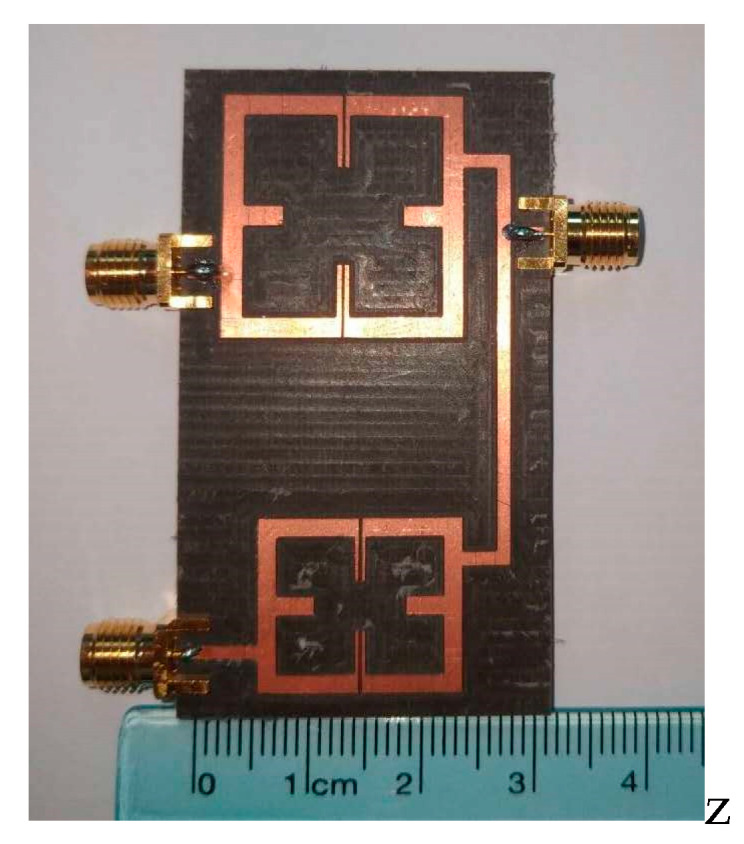
Actual production diagram of quad-channel diplexer.

**Figure 13:**
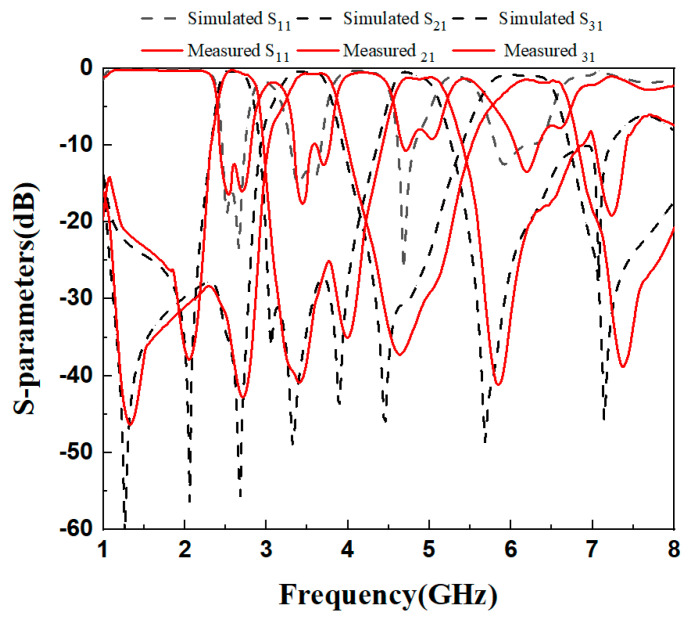
Comparison between simulation and actual measurement.

**Figure 14:**
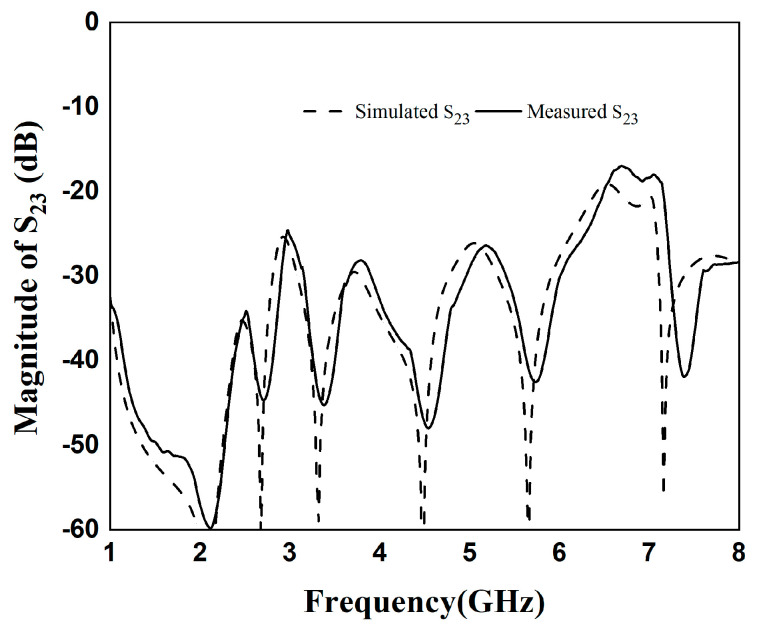
Comparison of simulated and measured S_23._

In the fourth step, the designed quad-channel diplexer is fabricated and tested. The designed diplexer is made of a 5880 substrate. And it was measured by the HP8510C network analyzer. Figure 12 shows the actual fabrication of the diplexer. The fabricated dimensions are as follows: L_1_ = 14.76 mm, L_2_ = 3.9 mm, Ls_1_ = 3.3 mm, W_1_ = 1.6 mm, W_2_ = 0.36 mm, g_1_ =0.2 mm; t_1_ = 4 mm, L_3_ = 19.45 mm, L_4_ = 4.7 mm, Ls_2_ = 3.5 mm, W_3_ = 1.8 mm, W_4_ = 0.45 mm, g_2_ = 0.2 mm, t_2_ = 5 mm, Lc_1_ = 32.1 mm, Lc_2_ = 10.1 mm, t = 7.1 mm. The actual dimensions of the diplexer are approximately 67 mm×28.3 mm, which is equivalent to 0.75λg × 0.32λg, where λg denotes the vacuum wavelength at the center frequency of 2.6 GHz. Figure 13 depicts the comparison between simulation and actual measurement. In the quad-channel diplexer, the upper dual-band filter attains that the center frequency of the first passband is 3.48 GHz, with a −3 dB bandwidth of 17.8%, and an average insertion loss |S_21_|= 0.47 dB. The center frequency of the second passband is 6.3 GHz, featuring a −3 dB bandwidth of 13.5%. The average insertion loss |S_21_|= 0.9 dB. The lower dual-band filter achieves a center frequency of the first passband of 2.6 GHz, with a −3 dB bandwidth of 14.6%, and an average insertion loss |S_21_|= 0.45 dB. The center frequency of the second passband is 4.8 GHz, and the −3 dB bandwidth is 13.5%. The average insertion loss |S_21_|= 0.5 dB. Owing to the adoption of 0-degree feed at the input and output ends, seven transmission zeros are generated at 2.05 GHz, 2.68 GHz, 3.05 GHz, 3.32 GHz, 3.88 GHz, 5.67 GHz, and 7.14 GHz, improving the high selectivity. Figure 13 shows good agreement, but slight shifts exist. Due to the relatively old engraving machine and the relatively thick engraving needle, as well as the fact that the measurement sensitivity to minor disturbances increases at higher frequencies, the deviation between the measured data and the simulated data becomes larger at higher frequencies. Concurrently, due to the favorable impedance matching, the |S_23_| > 20 dB between the upper dual-band filter and the lower dual-band filter ensures good isolation in Figure 14.

Table 1 shows the comparison between the diplexer designed in this paper and those designed in other literatures. It can be seen from Table 1 that the diplexer designed in this paper has lower insertion loss and more wider 3 dB bandwidth for the four passbands. Meanwhile, the diplexer designed in this paper has seven transmission zeros. At the edge of each passband, transmission zeros are generated, thereby enhancing the passband selectivity of the dual-band filter and the isolation of the diplexer.

## 4. Conclusions

This paper designs a quad-channel diplexer and implements it by using the simple-structured SLSIR. SLSIR uses a new impedance ratio K to control the resonant mode without increasing the overall circuit size. Two filters are electrically coupled through a pair of resonators to form a dual passband. Impedance matching was achieved by connecting two dual-band filters as input terminals through T-joint. The overall designed diplexer circuit has a simple structure and good performance.

## Figures and Tables

**Table 1:** Performance comparison with some reported quad-channel diplexers.

Literatures	1st/2nd/3rd/4th Passbands (GHz)	|S_21_| (dB)	FBW (%)	Passband Selectivity	Circuit Size (mm2)(λ^2^_0_)
7	0.9/1.29/2.41/3.5	1.2/ 0.9/1.2/1.09	12.78/20.88/8.5/9.96/	High	0.03396
9	2.12/2.45/3.90/4.48	2.10/2.82/1.95/2.6	5.66/6.32/6.35/5.13	Medium	0.0575
10	1.7/1.9/3/3/3.6	0.53/0.55/0.87/0.78	NA/NA/NA/NA	Medium	0.022575
12	1.85/2.45/2.15/3.50	1.7/1.2 /2.3/0.65	5.40/4.08/4.65/ 5.71	Medium	0.0704
15	1.50/1.83/2.32/2.92	1.55/1.21/1.54/1.28	7.3/7.5/6.0/5.4	High	0.2596
Proposed Filter	2.6/3.48/4.8/6.3	0.45/0.47/0.5/0.9	14.6/17.8/13.5/13.5	High	0.24

## Data Availability

All the material conducted in the study is mentioned in article.

## References

[1] Chen C.F., Huang T.Y., Chou C.P., Wu R.B. (2006). Microstrip diplexers design with common resonator sections for compact size, but high isolation. IEEE Trans. Microw. Theory Tech..

[2] Chinig A., Errkik A., El A.L., Tajmouati A., Zbitou J., Latrach M. (2016). Design of a microstrip diplexer and triplexer using open loop resonators. J. Microw. Optoelec. Electro. Appl..

[3] Chuang M.L., Wu M.T. (2011). Microstrip diplexer design using common T-shaped resonator. IEEE Microw. Wireless Compon. Lett..

[4] Tang H.J., Hong W., Chen J., Luo G.Q., Wu K. (2007). Development of millimeter-wave planar diplexers based on complementary characters of dual-mode substrate integrated waveguide filters with circular and elliptic cavities. IEEE Trans. Microw. Theory Techn..

[5] Chi P.L., Shih H.T., Yang T. (2021). 5G Millimeter-wave substrate-integrated waveguide quad-channel diplexer with high in-band and wideband isolation. IEEE Microw. Wireless Compon. Lett..

[6] Chinig A. (2018). Review on technologies used to design RF diplexers. Inter. J. Biosens. Bioelectron..

[7] Ji S.Y., Xu J., Zhou G.Q., Han B.Y. (2022). Compact quad-channel diplexer design using dual-band bandpass filters with multiple transmission zeros. Int. J. RF Microw. Comput. Aided. Eng..

[8] Ren B.P., Le C.F., Guan X.H., Ma Z.W. (2019). Short-circuited stub-embedded ring resonator and its application in diplexer. IEEE Access.

[9] Gorur A.K., Turkeli A., Buyuktuna M., Dogan E., Karpuz C., Gorur A. (2022). A high isolation quad-channel microstrip diplexer based on codirectional split ring resonators. Microw. Opt. Technol. Lett..

[10] Sobhan R., Salah I.Y., Yaqeen S.M., Muhammad A.C., Aqeel A.A., Afshin M., Saeed R. (2023). Design of a Compact Quad-Channel Microstrip Diplexer for Land S Band Applications. Micromachines.

[11] Soaad L.A., Raaed T.H. (2024). Multilayered Stub Loaded-SIR for Compact Dual-BPF and Quad-channel Diplexer Design. Radioengineering.

[12] Tantiviwat S., Ibrahim S.Z., Razalli M.S. (2019). Design of quad-channel diplexer and tri-Band bandpass filter based on multiple-mode stub-loaded resonators. Radioengineering.

[13] del Río J.L.M., Fernández-Prieto A., Martel J., Boix R.R., Medina F. (2022). Compact multilayered balanced-to-balanced dual-and tri-band diplexers based on magnetically coupled open-loop resonators. IEEE Access.

[14] Song K., Yao J., Chen Y., Zhou Y., Zhu Y., Fan Y. (2020). Balanced diplexer based on substrate integrated waveguide dual-mode resonator. IEEE Trans. Microw. Theory Techn..

[15] Wen T.L., Zhang H.R., Chai X.J., Mou J.C., Hei Y.Q., Shi X.W. (2023). Novel unbalanced-to-balanced quad-channel diplexer with controllable bandwidth and high isolation. IEEE Trans. Circuits Syst. II.

[16] Hong J.S. (2011). Microstrip Filters for RF/Microwave Applications.

